# Validity of the National Health Security Preparedness Index as a Predictor of Excess COVID-19 Mortality

**DOI:** 10.1017/S1049023X20001521

**Published:** 2021-01-05

**Authors:** Mark E. Keim, Alex P. Lovallo

**Affiliations:** 1. DisasterDoc LLC, Atlanta, Georgia USA; 2. Beth Israel Deaconess Medical Center Disaster Medicine Fellowship, Harvard University, Boston, Massachusetts USA; 3. Rollins School of Public Health, Emory University, Atlanta, Georgia USA

**Keywords:** coronavirus, health security, mortality, public health preparedness

## Abstract

**Objective:**

This study compared 2019 values for the National Health Security Preparedness Index (NHSPI) with 2020 rates of coronavirus disease 2019 (COVID-19)-related mortality as reported by the 50 US states and Puerto Rico during the first six months of the US pandemic (March 1 - August 31, 2020).

**Methods:**

Data regarding provisional death counts and estimates of excess deaths for COVID-19 according to state and territory were downloaded from the Centers for Disease Control and Prevention (CDC) National Center for Health Statistics website. Reporting included the six-month-long period of March 1 - August 31, 2020. Excess mortality rates were calculated as the number of excess deaths per 100,000 persons in each state population using 2019 US Census Bureau data. Mean values for state and territorial NHSPI domain indices were compared to state and territorial rates of COVID-19-related excess mortality using multiple linear regression, including analysis of variance. Correlations between the 51 state and territorial NHSPI values and corresponding COVID-19 excess mortality rates were calculated using Pearson’s correlation coefficient.

**Results:**

These calculations revealed a high degree of variance (adjusted r square = 0.02 and 0.25) and poor correlation (P = .16 and .08) among values for the overall NHSPI as compared to low and high estimates of excess COVID-19 mortality rates for 50 US states and Puerto Rico.

There was also a high degree of variance (adjusted r square = 0.001 and 0.03) and poor correlation (P values ranging from .09 to .94) for values for the six individual domains of the NHSPI as compared to low and high estimates of excess COVID-19 mortality rates for 50 US states and Puerto Rico.

**Conclusion:**

The NHSPI does not appear to be a valid predictor of excess COVID-19 mortality rates for 50 US states and Puerto Rico during the first six months of the pandemic.

## Introduction

Over the past 20 years, US local, state, and federal agencies have implemented a wide range of measures for public health emergency preparedness; “but these efforts have not resulted in a clear picture of the nation’s preparedness owing to ambiguous and uncertain preparedness goals, a lack of agreement about what the measures should aim at and how they should be interpreted, and a weak system of accountability for producing results.”^1,2^

Health security is a recent concept that encompasses “activities and measures across sovereign boundaries that mitigates public health incidents to ensure the health of populations.”^3^ However, like preparedness, there remains limited consensus regarding the definition and scope of health security.^4^

In 2012, the US Centers for Disease Control and Prevention (CDC; Atlanta, Georgia USA) initiated development of the National Health Security Preparedness Index (NHSPI) for “measuring the nation’s progress in preparing for, responding to, and recovering from disasters and other large-scale emergencies that pose risks to health and well-being in the United States.”^5^

As a measurement tool, the NHSPI is intended to “summarize levels of preparedness achieved within individual states and for the nation as a whole” and has been used for this purpose. Health impact is implied as the domains of the NHSPI are reportedly considered to have been “shown to be important in protecting people from the health consequences of disasters and other large-scale hazards and emergencies.”^5^

Validity is central to determining the utility of any scale. Validity describes a test’s ability to produce results consistent with other measures of the same characteristic and it requires external criteria.^6^ Predictive validity is considered to be achieved when there is significant correlation between an experimental test (eg, the NHSPI) and a reference criterion standard (eg, excess coronavirus disease 2019 [COVID-19] mortality). This study evaluated the validity of the NHSPI for predicting excess COVID-19-related mortality.

## Methods

This study compared 2019 values for the NHSPI with rates of COVID-19-related morbidity and mortality as reported by the 50 US states and Puerto Rico during the first six months of the pandemic (March 1 - August 31, 2020).

### Data Collection

State data summaries for the 2019 NHSPI were downloaded from the 2019 Preparedness Index Data Preview website.^7^ Table 1 lists the 2019 NHSPI indicators grouped into six domains and 19 sub-domains.

**Table 1.** Domains and Sub-Domains of the NHSPI

Domain 1: Health Security Surveillance
Sub-Domain 1.1: Health Surveillance & Epidemiological Investigation
Sub-Domain 1.2: Biological Monitoring & Laboratory Testing
**Domain 2: Community Planning & Engagement Coordination**
Sub-Domain 2.1: Cross-Sector/Community Collaboration
Sub-Domain 2.2: Children & Other At-Risk Populations
Sub-Domain 2.3: Management of Volunteers during Emergencies
Sub-Domain 2.4: Social Capital & Cohesion
**Domain 3: Incident & Information Management**
Sub-Domain 3.1: Incident Management
Sub-Domain 3.2: Information Management
**Domain 4: Health Care Delivery**
Sub-Domain 4.1: Prehospital Care
Sub-Domain 4.2: Hospital and Physician Services
Sub-Domain 4.3: Long-Term Care
Sub-Domain 4.4: Mental & Behavioral Health Care
Sub-Domain 4.5: Home Care
**Domain 5: Countermeasure Management**
Sub-Domain 5.1: Medical Materiel Management, Distribution, & Dispensing
Sub-Domain 5.2: Countermeasure Utilization & Effectiveness
**Domain 6: Environmental & Occupational Health**
Sub-Domain 6.1: Food & Water Security
Sub-Domain 6.2: Environmental Monitoring
Sub-Domain 6.3: Physical Environment and Infrastructure
Sub-Domain 6.4: Workforce Resiliency

Abbreviation: NHSPI, National Health Security Preparedness Index.

Data regarding provisional death counts and excess deaths for COVID-19 (according to state and territory) were downloaded from the CDC National Center for Health Statistics website.^8^ Reports were downloaded for the six-month-long period of March 1 - August 31, 2020. The CDC defined excess deaths as the difference between the observed numbers of deaths in specific time periods and expected numbers of deaths in the same time periods. Estimates of excess deaths were calculated by CDC using Farrington surveillance algorithms.^8^ The lower and upper estimates of excess mortality were calculated as the difference between the observed count and one of two thresholds (either the average expected count or the upper bound of the 95% prediction interval) by jurisdiction. Excess mortality rates were calculated as the number of excess deaths per 100,000 persons in each state population using 2019 US Census Bureau (Suitland, Maryland USA) data.^9^

### Data Analysis

Mean values for state and territorial NHSPI domain indices were compared to state and territorial rates of COVID-19-related excess mortality using multiple linear regression, including analysis of variance. Correlations between the 51 (state and territorial) NHSPI values and corresponding COVID-19 excess mortality rates were calculated using Pearson’s correlation coefficient.

## Results

In general, these calculations failed to demonstrate any significant association between values of the overall NHSPI (or its six domains) and excess COVID-19 mortality rates for 50 US states and Puerto Rico.

Table 2 reveals a high degree of variance and poor correlation of values for the overall NHSPI as compared to low and high estimates of excess COVID-19 mortality rates for 50 US states and Puerto Rico.

**Table 2.** Linear Regression Statistics for 2019 Values of the Overall NHSPI, as Compared to Low and High Estimates of Excess COVID-19 Mortality Rates for 50 US States and Puerto Rico

	Adjusted R Square	Standard Error	Significance F	T Statistic	P Value	95% Confidence Intervals
Low Estimate of Excess COVID-19 Mortality Rate	0.02	38.05	0.16	1.42	.16	-9.31 to 54.25
High Estimate of Excess COVID-19 Mortality Rate	0.25	40.86	3.13	1.77	.08	-4.07 to 64.20

Abbreviation: NHSPI, National Health Security Preparedness Index.

Table 3 also indicates a high degree of variance and poor correlation of values for the six individual domains of the NHSPI as compared to low and high estimates of excess COVID-19 mortality rates for 50 US states and Puerto Rico.

**Table 3.** Linear Regression Statistics for NHSPI Domains, as Compared to Low and High Estimates of Excess COVID-19 Mortality Rates for 50 US States and Puerto Rico

Domain	Estimate Range	Adjusted R Square	Standard Error	Significance F	T Statistic	P Value	95% Confidence Intervals
1.0 Health Security Surveillance	Low	0.001	38.45	0.44	-0.36	.72	-20.22 to 14.13
	High	0.03	41.11	0.30	0.08	.94	-19.09 to 17.65
2.0 Community Planning & Engagement Coordination	Low	0.001	38.45	0.44	1.37	.18	-5.04 to 26.60
	High	0.03	41.11	0.30	1.71	.09	-2.53 to 31.31
3.0 Incident & Information Management	Low	0.001	38.45	0.44	-0.44	.66	-25.90 to 16.58
	High	0.03	41.11	0.30	-0.78	.44	-31.50 to 13.92
4.0 Health Care Delivery	Low	0.001	38.45	0.44	0.51	.61	-13.90 to 23.41
	High	0.03	41.11	0.30	1.10	.28	-9.10 to 30.79
5.0 Countermeasure Management	Low	0.001	38.45	0.44	-0.08	.94	-16.29 to 17.07
	High	0.03	41.11	0.30	-0.07	.94	-17.37 to 16.17
6.0 Environmental and Occupational Health	Low	0.001	38.45	0.44	1.49	.14	-4.44 to 29.51
	High	0.03	41.11	0.30	0.94	.35	-9.66 to 26.64

Abbreviation: NHSPI, National Health Security Preparedness Index.

## Discussion

A research-practice gap exists across all fields of public health, including disaster-related health science.^10,11^ Public health has moved forward in recent years to bridge this gap. Evidence-based public health calls for knowledge of the determinants and consequences of disease, as well as the efficacy, effectiveness, and costs of interventions.^12,13^ And yet, despite repeated urging of public health leadership, disaster epidemiology remains chiefly concerned with etiological, rather than evaluative, hypotheses.^14,15^

Scale development and validation are critical to much of the work in public health. However, scale development is not a straightforward endeavor. There are many steps to scale development. There is significant jargon within these techniques. The work can be costly, time consuming, and complex statistical analysis is often required. Despite the availability of a large amount of technical literature on scale theory and development, many incomplete scales remain.^16^

Part of the challenge in establishing a valid scale for preparedness stems from the reliance upon expert opinion as compared to a set of specific empirical observations to reach the overarching conclusion.

Future developments are not always predicted correctly by Delphi consensus, especially those involving complex forecasts (eg, disasters) with multiple factors (eg, 140 separate measures). The Delphi method is therefore used most successfully in forecasting single scalar indicators.

Practical application of the NHSPI is unlikely to occur if it is not directly tied to indicators of health outcome (ie, mortality). This challenge is compounded by the polysemous nature of both of the key phrases in question, “preparedness” and “health security,” making predicted health outcomes difficult to define, let alone measure. There would appear to be limited practical utility for a health-related index that has no proven association with actual health outcomes.

## Study Limitations

While this study does raise an important and reportable question regarding the criterion-related validity of the NHSPI, the investigation does not, however, prove the null hypothesis. Further study is necessary to further detail the association between the NHSPI and COVID-19-related mortality. Subsequent studies should also include a systematic analysis of content validity considering the relatively ambiguous and polysemous set of definitions associated with terms such as “health security” and “preparedness.”

## Conclusion

The NHSPI does not appear to be a valid predictor of excess COVID-19 mortality rates for 50 US states and Puerto Rico during the first six months of the pandemic.
